# Gut Microbiota and Metabolic Syndrome: Relationships and Opportunities for New Therapeutic Strategies

**DOI:** 10.1155/2024/4222083

**Published:** 2024-07-15

**Authors:** Emmanuel Henry Ezenabor, Aishat Abimbola Adeyemi, Oluyomi Stephen Adeyemi

**Affiliations:** Department of Biochemistry Medicinal Biochemistry, Nanomedicine & Toxicology Laboratory Bowen University, Iwo 232102, Osun State, Nigeria

## Abstract

Since its discovery, numerous studies have shown the role of the microbiota in well-being and disease. The gut microbiota represents an essential factor that plays a multidirectional role that affects not just the gut but also other parts of the body, including the brain, endocrine system, humoral system, immune system, and metabolic pathways, as well as host-microbiome interactions. Through a comprehensive analysis of existing literature using the desktop research methodology, this review elucidates the mechanisms by which gut microbiota dysbiosis contributes to metabolic dysfunction, including obesity, dyslipidaemia, hypertension, atherosclerosis, hyperuricemia, and hyperglycaemia. Furthermore, it examines the bidirectional communication pathways between gut microbiota and host metabolism, highlighting the role of microbial-derived metabolites, immune modulation, and gut barrier integrity in shaping metabolic homeostasis. Importantly, the review identifies promising therapeutic strategies targeting the gut microbiota as potential interventions for metabolic syndrome, including probiotics, prebiotics, symbiotics, dietary modifications, and faecal microbiota transplantation. By delineating the bidirectional interactions between gut microbiota and metabolic syndrome, the review not only advances our understanding of disease pathophysiology but also underscores the potential for innovative microbiota-based interventions to mitigate the global burden of metabolic syndrome and its associated complications.

## 1. Introduction

Gut microbiota, otherwise called “intestinal microbiota” or “gut microflora,” refers to a vast, complex collection of microorganisms that have a great impact on human health. It comprises trillions of microorganisms, including bacteria, viruses, fungi, and archaea, which inhabit various regions of the digestive tract, such as the stomach, small intestine, and predominantly the colon [1]. In the colon, they are comprised mainly of anaerobic bacterial species. The relationship between humans and these bacteria is symbiotic, as both species benefit in a variety of ways. Humans provide the bacteria with nutrients and an anaerobic home in our colon, while they provide a range of benefits. These microorganisms perform a myriad of functions vital to human health, such as aiding in digestion, synthesizing essential vitamins, and interacting with the immune system [2]. Additionally, the balance of microorganisms in the gastrointestinal tract is largely maintained by the gut microbiota. Changes in the makeup of the gut microbiota have drawn much attention because they can prevent the overgrowth of potentially harmful pathogens and maintain the integrity of the gut barrier in the context of metabolic syndrome. Individuals with metabolic syndrome have been shown to have changes in the diversity and abundance of particular microbial species [3].

The amount and makeup of microorganisms in the gastrointestinal tract vary widely between individuals because the gut microbiota is highly individualized. A number of factors, including environmental, genetic, and lifestyle ones, contribute to the uniqueness of the gut microbiota. The functions of the gut microbiota can be impacted by disturbances in their composition. Three kingdoms of life on Earth—archaea, bacteria, and eukarya—are represented in the human gut. In this ecosystem, which is dominated by bacteria, two of the ten known phyla of bacteria—the *Firmicutes* and the *Bacteroidetes*—make up more than 90% of the phylotypes. Apart from the dominant phyla, there also exist *Actonibacteria*, *Cyanobacteria*, *Fusobacteria, Lentispaerae*, and *Proteobacteria* [4]. Recent studies have changed our understanding of human health by illuminating the complex interactions between metabolic syndrome disorders and the gut microbiota [5]. A large number of microorganisms in the digestive tract make up the gut microbiota, which has become important in controlling metabolic processes and the emergence of metabolic disorders [6].

An imbalance in the relative abundance of various microbial species is known as dysbiosis, and it has been shown to influence inflammation, insulin resistance, and obesity. Disturbances in metabolic homeostasis have been associated with certain microbial imbalances, including an elevated ratio of *Firmicutes* to *Bacteroidetes* [7]. Additionally, the gut microbiota actively participates in the breakdown of dietary fibres, resulting in the production of butyrate, propionate, and acetate, which are short-chain fatty acids (SCFAs). These metabolites from microorganisms have a significant impact on host metabolism, affecting insulin sensitivity, inflammation, and energy homeostasis. Interestingly, it has been shown that SCFAs influence how adipose tissue functions and how the body uses glucose [8]. By examining the complex relationships between gut microbiota and metabolic syndrome, this review seeks to illuminate the underlying mechanisms and possible treatment options for the amelioration of metabolic syndrome.

## 2. Methods

The study utilized a desktop research methodology, a comprehensive approach that involves gathering secondary data or information readily available without the need for fieldwork. This methodology relies on existing resources, making it a cost-effective alternative to traditional field research, with primary expenses typically allocated to personnel time, telecommunications, and reference materials. Articles published in English between 2000 and 2023 were identified and extracted using specific search terms, such as “gut microbiota mechanism of influence,” “gut microbiota mechanism and metabolic syndrome,” and “therapeutic opportunities in gut microbiota.” The secondary data were accessible through online repositories and academic libraries, such as PubMed and Google Scholar, facilitating the identification of relevant scholarly literature. While 143 articles were initially retrieved, 42 were excluded due to limited access to full texts. Furthermore, manual scrutiny of eligible studies was conducted to scrutinize their reference lists, thereby uncovering original and supplementary sources pertaining to the mechanisms of action of gut microbiota.

## 3. Gut Microbiota and Its Mechanisms of Actions

**Figure 1:**
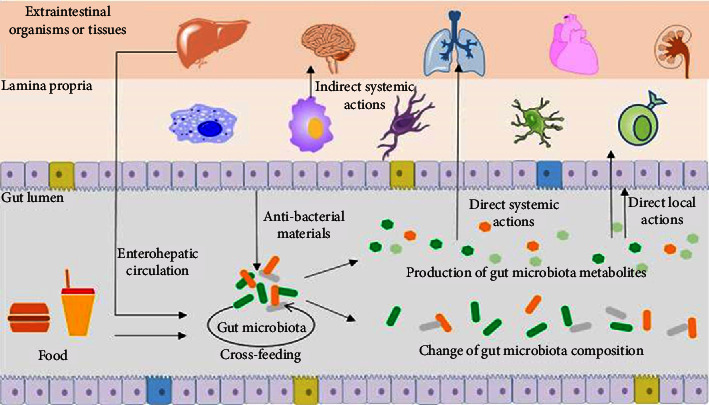
Gut microbiota mechanism of influence. The figure delineates the influence of dietary intake on the composition of gut microbiota, whose mechanisms exert effects extending to multiple organs within the body, encompassing the brain, kidneys, heart, and lungs. These effects culminate in a spectrum of maladies, including neurodegenerative disorders and pneumonia in the brain, heart failure and atherosclerosis in the cardiovascular system, lung cancer and asthma in pulmonary function, and metabolic disorders, such as obesity and diabetes. Source: Lui et al. [8].

Metabolites of the gut microbiota can affect hosts' functions directly or indirectly in a variety of ways, as seen in Figure 1. Human health and disease are significantly influenced by the makeup of the gut microbiota, and there is a close association between the gut microbiota's composition and metabolism. The metabolites of the gut microbiota can directly or indirectly control the function and composition of the gut microbiota by targeting either the gut bacteria or the hosts. For instance, the gut microbiota can utilize short-chain fatty acids (SCFAs) as an energy source, and certain gut bacteria can be inhibited from growing when SCFA concentrations are high [8]. Furthermore, in order to stop infections from invading, SCFAs have the ability to control the synthesis of secretory immunoglobulin A, a noninflammatory antibody produced by hosts [9].

In turn, gut microbiota metabolites have the ability to modify their own composition, which in turn affects the hosts' functions indirectly. Metabolites from the gut microbiota can also directly affect host targets located near or far from the gastrointestinal tract. For instance, intestinal epithelial cells directly sense SCFAs when they are released into the gastrointestinal tract and use this information to affect the operation of the gut barrier [10]. Moreover, SCFAs can be administered to distant organs and tissues, where they are directly sensed by the target organs and tissues and cause significant physiological alterations in the hosts. Other metabolites of the gut microbiota, in addition to SCFAs, can also act directly or indirectly on targets located within, close to, or outside of the digestive tract [11]. The phenomenon wherein one bacterium assimilates or exchanges bacterial metabolites with another is termed “bacterial cross-feeding” [12]. A typical product of bacterial cross-feeding is lactate, which serves as an end product of Bifidobacterium metabolism. Under conditions of pure starch, the growth of *Eubacterium hallii* is impeded. However, cocultivation of *E. hallii* with *Bifidobacterium adolescentis* leads to a significant reduction in lactate concentration and a concurrent increase in butyrate concentration. This indicates that *E. hallii* can utilize lactate released by *B. adolescentis* to synthesize butyric acid. The process of cross-feeding enhances the nutritional status of bacteria and alters their susceptibility to antibiotics, consequently influencing the composition and functionality of the gut microbiota [11].

### 3.1. Gut Microbiota and Metabolic Syndrome

Metabolic syndromes refer to a complex number of metabolic abnormalities that raise the risk of cardiovascular diseases significantly, type 2 diabetes, and other health complications [7]. Some examples of metabolic syndrome include obesity, hyperglycaemia, hyperuricemia, atherosclerosis, hypertension, and dyslipidaemia [13].

### 3.2. Gut Microbiota and Obesity

Excess accumulation of body fat is a characteristic feature of obesity. The main cause of obesity is a prolonged energy imbalance between consumed calories and expended calories [13]. A number of hereditary and nongenetic (environmental) factors cause obesity. Obesity, as defined by the World Health Organization, is a body mass index (BMI) of more than 30 kg/m^2^, yet national definitions differ. For instance, a BMI of 28 kg/m^2^ or higher is deemed obese in China. A thorough investigation reveals that 10% of people worldwide are fat, and about a third are overweight [14]. Obesity has become a major global health issue due to its high risk of health complications. In addition to causing changes in appearance, obesity is linked to oxidative stress, chronic inflammation, abnormal glucose, and lipid metabolism, and an even higher risk of a number of diseases, including diabetes, cancer, and cardiovascular disease [15]. Growing research in recent years has suggested that an imbalance in the gut microbiota may play a role in the causation of obesity [16].

The initial indication of a correlation between obesity and the gut microbiota emerged from studies conducted on germ-free mice. These investigations demonstrated that gut microbes have the capability to stimulate adipose tissue expansion in the host, as evidenced by the transplantation of gut microorganisms from conventionally raised mice into germ-free animals. This transfer resulted in heightened adiposity and insulin resistance levels in the recipients, despite a reduction in food intake. Additionally, 16S ribosomal ribonucleic acid gene sequencing has implicated two predominant bacterial phyla, Firmicutes and Bacteroidetes, in the context of obesity. Specifically, analysis of the gut microbiota in obese mice revealed a notable reduction in Bacteroidetes abundance by 50%, accompanied by a corresponding elevation in Firmicutes levels [17].

Hard-to-digest polysaccharides are fermented by the gut microbiota into short-chain fatty acids (SCFAs), which are either absorbed by the gut or egested in stool. SCFAs are essential for maintaining energy balance. The primary constituents of SCFAs are butyrate, propionate, and acetate. By producing glucagon-like peptide-1, peptide YY, and other intestinal hormones, acetate can confer positive effects on the host's energy metabolism. It can also boost lipid oxidation, lower levels of proinflammatory cytokines, and lipolysis in the system, and promote energy consumption [18]. Using the AMPK/LSD1 pathway, propionate encourages intestinal lipolysis and energy balance in mice [19]. The primary energy source for the colon is butyrate, which is oxidized to provide most of the energy needed by intestinal epithelial cells. By increasing the amount of butyrate produced, the gut microbiota's butyrate-producing bacteria improve lipid metabolism through the butyrate-SESN2/CRTC2 pathway. On the other hand, a high content of butyrate can counteract the metabolic effects and lower the probiotic proportion [20].

Furthermore, studies showed that gut bacteria could control how much fat is stored in the body [21, 22]. The host's gut microbiota facilitates glucose uptake in the intestine and serum glucose levels, which in turn raises the expression levels of two fundamental transcription factors that cause the liver to produce fat: sterol regulatory element binding protein and carbohydrate response element binding protein. Triglycerides absorbed by fat cells in the liver are transported into the bloodstream with the assistance of lipoprotein lipase [23]. Nevertheless, it has been demonstrated that the gut microbiota influences host circadian rhythms in a diet-dependent way [8]. The incidence of obesity may rise because of the disruption of the circadian rhythm. Through the regulation of the circadian transcription factor NFIL3, microorganisms control the intake and storage of lipids. An essential biological pathway for the interaction of the microbiota with the circadian clock is the ILC3-STAT3 signalling pathway. According to a recent study, the gut microbiota induces rhythmic histone acetylation in intestinal epithelial cells by expressing histone deacetylase 3, which causes the transcription of the lipid transporter gene, Cd36, to become rhythmic. This facilitates obesity and lipid absorption [24]. Mice devoid of germs were bred with the excrement of both jet lag sufferers and normal schedulers. The transplanted mice that suffered from jet lag consequently became obese and insulin-resistant. Jet lag causes microbial dysbiosis due to disrupted eating cycles, which encourages glucose intolerance and obesity [25]. While it is possible to identify distinct microorganisms in obese versus normal individuals and confirm the involvement of bacteria in obesity, this deserves greater consideration. Obesity is caused by changes in the gut microbiota that impact hunger, energy intake, circadian rhythm, and chronic inflammation. Thus, one method of treating obesity is the focused reconstruction of the gut microbiota structure, for example, via faecal bacterial transplantation.

### 3.3. Gut Microbiota and Dyslipidaemia

Dyslipidaemia is a metabolic syndrome usually associated with aberrant lipid profiles, defined by raised triglycerides, reduced high-density lipoprotein cholesterol, and an increased prevalence of tiny, dense low-density lipoprotein particles [26]. Stroke, atherosclerosis, coronary heart disease, and other cardio-cerebrovascular aberrations can be brought on by dyslipidaemia. Its occurrence has been rising for a number of years, and its primary cause is the combination of hereditary and environmental variables. Numerous studies have demonstrated a direct relationship between dyslipidaemia development and gut microbiota disorders. By controlling the equilibrium of cholesterol, its metabolites, particularly bile acids, trimethylamine N-oxide, and short-chain fatty acids, have an impact on dyslipidaemia. According to a recent study, dyslipidaemia's onset and progression are strongly correlated with alterations in the gut microbiota's structure [27].

The incidence and progression of dyslipidaemia can be made better or worse by changing the amount of SCFA in the body [28]. Once produced, intestinal epithelial cells quickly absorb and catabolize SCFAs. The majority of acetate either is utilized by adipocytes for lipogenesis or is oxidized by muscle, avoiding the splanchnic circulation [28, 29]. Conversely, the parasympathetic nervous system might be activated by higher gut microbiota production of acetate, which would then promote glucose-stimulated insulin secretion, increase hunger, and ultimately result in unbalanced lipid metabolism [30]. Propionate decreases calorie intake by inducing the secretion of glucagon-like peptide 1 and the satiety hormone peptide YY (PYY) in the intestine. The pancreas secretes insulin in response to stimulation from PYY and GLP1. Insulin enters the bloodstream, encourages the breakdown of glucose into fat, increases the synthesis of fat, prevents the breakdown of fat, and exacerbates dyslipidaemia. The liver's uptake of acetate and propionate served as substrates for the processes of gluconeogenesis and lipogenesis, which enhanced energy dissipation. Furthermore, via brain-enteric nerve connections, propionate can also indirectly stimulate intestinal gluconeogenesis. In summary, elevated propionate levels may raise the risk of dyslipidaemia [31].

The gut microbiota also affects dyslipidaemia by controlling the enterohepatic circulation of bile acid, which interferes with the metabolism of cholesterol. Complementary methods were used to measure hepatic cholesterol synthesis, plasma cholesterol levels, and enterohepatic circulation. The results showed that the gut microbiota is a strong regulator of hepatic cholesterol synthesis, plasma cholesterol levels, and enterohepatic circulation and that the composition of the gut microbiota affects cholesterol biosynthesis, absorption, and circulating cholesterol levels [32]. Bile acids are novel metabolic regulators with lipid-digesting properties [33]. Treatment for dyslipidaemia mostly focuses on regulating the gut microbiota and bile acid metabolites. Modifications in microbiome composition may affect the management of metabolic disorders. Due to the gut microbiota's defensive mechanisms against bile acid toxicity, cholesterol and bile acids undergo a series of chemical alterations that influence their signalling mechanisms, which can lead to the destruction of gut bacteria [34]. In the colon and terminal ileum, the gut microbiota influences the conversion of primary bile acids (BAs) to secondary BAs through the action of microbe-associated bile-salt hydrolase and 7-dehydroxylase. Failure of this conversion process leads to a deficiency of secondary BAs and can predispose individuals to *Clostridium difficile* infection, resulting in severe intestinal inflammation [35]. The gut microbiota plays a crucial role in regulating lipid metabolism by maintaining balance in the bile acid pool and its composition. Bile acids act as ligands for TGR5 and FXR, enabling their activation and subsequent modulation of lipid metabolism, thereby impacting blood lipid levels and contributing to the regulation of lipid and glucose metabolism [36].

Additionally, by controlling the trimethylamine/flavin-containing monooxygenase 3/trimethylamine N-oxide (TMA/FMO3/TMAO) pathway, influencing bile acid metabolism and reverse cholesterol transcription, controlling cholesterol levels, and influencing blood lipid, the gut microbiota interferes with lipid metabolism [37]. Research has demonstrated that sex hormones, food, energy consumption, transcription regulator CCAAT, and enhancer binding protein can all affect the expression or function of liver FMO3. Moreover, it has been discovered that nuclear FXR, which is involved in bile acid metabolism in the liver and small intestine, controls FMO3. By controlling several processes related to liver lipogenesis and gluconeogenesis, intestinal cholesterol production, and macrophage-specific RCT, FMO3 may contribute to dyslipidaemia by upsetting cholesterol homeostasis [38]. Research indicates a mandatory function of the gut microbiota in the conversion of dietary phosphatidylcholine to the pro-atherosclerotic molecule TMAO. In this particular context, choline, which comprises a portion of trimethylammonium, is directly transformed into TMA by gut bacteria, after which TMAO is metabolized into the bloodstream via liver FMO3. TMAO has the ability to modulate macrophage receptors, decrease the expression of some bile acid transport genes in the liver (CYP7A1 and CYP27A1), disrupt RCT, and control cholesterol balance, all of which have an impact on blood lipids [39].

### 3.4. Gut Microbiota and Hypertension

Hypertension is a condition of elevated blood pressure, and it is a common feature of metabolic syndrome. The mechanisms linking hypertension to metabolic syndrome involve insulin resistance, sympathetic nervous system activation, and endothelial dysfunction [40]. The greatest significant modifiable risk factor for cardiovascular disease is hypertension. While genetic and lifestyle factors are assumed to have a combined role in the occurrence of hypertension, genome-wide association studies have demonstrated that genetics accounts for only a minor (<5%) fraction of the disease's incidence. On the other hand, lifestyle tends to have a significantly greater impact; for example, different lifestyle factors like salt intake and body mass index (BMI) can affect blood pressure readings by up to 5 mmHg [41]. Numerous cross-sectional studies conducted on people have evaluated correlations between blood pressure, or hypertension, and the composition of the gut microbiota. Certain findings about the composition of the microbiota and microbial alpha diversity are similar across studies, despite variations in sequencing techniques and downstream analyses. In nearly all of the research, decreased gut microbiota alpha diversity was linked to higher blood pressure [42].

The consumption of salt has an influence on the composition of the gut microbiota and the prevalence of hypertension. Increased use of salt has been linked in a number of animal models to a change in the makeup of the microbiota, with a decrease in *Lactobacillus* and *Oscillibacter* and an increase in *Lachnospiraceae*, *Ruminococcus*, and *Parasutterella* spp. [43]. Supplementing *Lactobacillus* spp. in a mouse model has been demonstrated to diminish salt-sensitive hypertension, likely through regulation of Th17 cells. The abundance of *Lactobacillus* has been linked to salt sensitivity in hypertension. Numerous other animal experiments verified *Lactobacillus* ability to reduce blood pressure [44]. However, in humans, only one of the cross-sectional investigations in hypertension people found a decrease in *Lactobacillus* spp. [42].

Studies using SCFAs as a human intervention to lower blood pressure have not produced any results. Butyrate did, however, appear to reduce blood pressure in individuals with metabolic syndrome who participated in intervention trials [45]. Furthermore, it has been shown that the Mediterranean diet, which raises SCFA levels, lowers blood pressure [46]. The varying effects of SCFA receptors may account for the association between SCFAs and both greater and lower blood pressure in animal models. Fatty acid receptor (FFAR)-3 and (FFAR)-2 (formerly known as GPR41 and GPR43) are two of the identified SCFA receptors [39]. Research conducted on animals has demonstrated that the impact of SCFAs on blood pressure varies based on the specific receptors they interact with, including the renal arteries, express FFAR2, which induces vasodilation in response to SCFAs [47].

Additionally, gut microbiota can affect gut permeability, which in turn affects how much endotoxins and metabolites are absorbed. The intestinal epithelium's barrier is mainly made up of enterocyte brush boundaries, which allow more hydrophobic substances to pass through than water-soluble ones. Furthermore, an additional route for paracellular absorption is facilitated by intercellular connections located at the lateral borders of enterocytes. These connections consist of adherens junctions on the basolateral side and tight junctions on the apical side, forming dynamic structures that regulate paracellular permeability [48]. The zonulin pathway, in conjunction with dietary factors, can influence the extent of this permeability. Zonulin, secreted by the basal lamina of the intestinal epithelium, binds to enterocytes, initiating a complex intracellular signalling cascade that ultimately phosphorylates tight junction proteins, thereby inducing permeability of the paracellular pathway [49]. *Vibrio cholerae* and other gut bacteria seem to take advantage of this physiological mechanism by secreting a toxin called zona occludens toxin, which is a homolog of zonulin and has comparable effects [50]. Based on animal studies, hypertension is associated with increased intestinal permeability. Gap junction protein mRNA levels were decreased in hypertensive rats, suggesting increased gut permeability. This was corrected when transplanting control faecal microbiota [51].

### 3.5. Gut Microbiota and Atherosclerosis

Atherosclerosis is the rapid build-up of plaques in the medium- or large-artery subendothelium. The process is brought on by the build-up of cholesterol in the artery's intimal layer, primarily in the form of low-density lipoproteins [52]. Blood pressure, vascular ageing, inflammation, and lipid metabolism are important factors in the complex process of atherosclerosis. Artery stiffness, resulting from the thickening of artery walls and the loss of elastic fibres, is intimately associated with atherosclerosis. An older population often has more arterial stiffness, which leads to a less flexible arterial system and a faster pulse wave. The development of the following atherosclerotic plaques is aggravated by the ensuing increased shear stress [53]. This process involves the build-up of cholesterol in artery walls, which triggers the phagocytic absorption of lipid particles by macrophages, transforming them into foam cells. Lipid oxidation leads to the crystallization of cholesterol, the release of proinflammatory cytokines, such as IL-1B and TNF-alpha, and the activation of the inflammasome. Statins have been shown to be beneficial in reducing atherosclerotic events because of their anti-inflammatory properties as well as their ability to reduce low-density lipoprotein cholesterol [54]. It has been demonstrated that an atherosclerotic plaque is a microbial ecosystem unto itself, housing microorganisms, such as *Chlamydia pneumoniae*, *Veillonella* spp., *Pseudomonas*, *Klebsiella*, and *Streptococcus*. Alternatively, by producing pro-atherogenic chemicals, the gut microbiota may have indirect pro-atherogenic effects. These metabolites may also contain the metabolites, such as SCFAs, that are linked to hypertension [55].

Trimethylamine (TMA) and TMAO have been widely studied in relation to the development of atherosclerosis. TMA is generated during the breakdown of substances like choline, carnitine, and lecithin that are present in foods like meat and eggs and are mostly created by gut microbes belonging to the families Clostridia and Enterobacteriaceae [56]. The enzyme flavin monooxygenase (FMO)-3, produced by the liver, oxidizes TMA into trimethylamine-N-oxide (TMAO) after absorption [57]. It has been shown that TMAO causes hyperreactivity of the platelets, which can promote thrombosis and hence atherosclerotic thrombotic events. In fact, TMAO administration increased atherosclerosis in a number of animal models [58].

Furthermore, evidence suggests that alterations in the gut microbiota influence bile acid metabolism and can impact conditions, such as hyperinsulinemia and inflammatory bowel disease. Primary bile acids are synthesized by the liver through the conversion of lipophilic cholesterol to lipophobic primary bile acids. Subsequently, these bile acids are excreted by the gall bladder and reabsorbed in the terminal ileum via bile acid transporters that rely on sodium [59]. Although only a small portion of bile acids reaches the large intestine, microbial modifications occur, including deconjugation, 7*α*-dehydroxylation, and 7*α*-hydrogenation, leading to the conversion of primary bile acids into secondary bile acids. Due to their hydrophobic nature, colonocytes can readily absorb secondary bile acids and transport them into the systemic circulation. It is estimated that approximately 5% of bile acids can bypass the enterohepatic cycle for elimination [60]. The G protein-coupled bile acid receptor TGR5 is expressed in various cell types, including leukocytes, macrophages, endothelial cells, and multiple organs, such as the liver, gall bladder, intestines, kidneys, pancreas, muscle, and adipose tissue [61]. Studies have shown that a TGR5 agonist (INT-777) possesses immunosuppressive properties, including the reduction of macrophage production of proinflammatory cytokines and attenuation of atherosclerotic plaque formation [62].

### 3.6. Gut Microbiota and Hyperuricemia

Hyperuricemia is characterized by high amounts of serum uric acid, often higher than 6 mg/dL for women and 7 mg/dL for men. A common condition brought on by reduced uric acid excretion or purine metabolism that affects individuals of all ages and genders. Gout is the most typical symptom of hyperuricemia. A metabolic disease known as hyperuricemia occurs when the limit solubility of 6.0 mg/dL of uric acid is exceeded in the serum [63]. Due to alterations in dietary habits and lifestyle, the occurrence of hyperuricemia has risen dramatically globally. The prevalence of hyperuricemia is higher in coastal urban areas, such as Thailand (10.6%) [64], the USA (21.0%), China (13.0%) [65], and Japan (20–25%) [66], while it is lower in Middle Eastern nations such as Saudi Arabia, Turkey, and Iran [67].

Frequent and excessive consumption of purine-rich meals can raise serum uric acid levels, which may raise the risk of hyperuricemia because uric acid is the final oxidation product of purine (adenine and guanine) metabolism [68]. It has been documented that gut bacteria play a role in purine oxidative metabolism. For example, *E. coli* can secrete xanthine dehydrogenase, a significant rate-limiting enzyme involved in the oxidative metabolism of purines, in the human gut [69]. Similarly, bacteria belonging to the genus Proteus can secrete xanthine dehydrogenase, which can convert purines to uric acid [70]. Additionally, *Lactobacillus* can reduce the intestinal uptake of purines, limiting increases in serum uric acid and exacerbating hyperuricemia [71]. The gut microbiota also facilitates the breakdown of purines and uric acid by secreting active enzymes. *Lactobacillus* and *Pseudomonas* can synthesize the enzymes uricase, allantoinase, and allantoicase, which can break down uric acid into 5-hydroxyisourate, allantoin, allantoate, and finally urea [72]. Overall, by encouraging the breakdown of purines and uric acid and decreasing their absorption in the intestinal system, the gut microbiota plays a significant role in minimizing the worsening of hyperglycaemia.

When gout patients were compared to healthy controls, a study that combined investigation of the microbiome and metabolome revealed that both the faecal microbiota and metabolites were simultaneously disrupted. A-ketoisocaproate, valine, phenylalanine, and citrulline are downregulated, while glucose, acetate, succinate, and certain amino acids are upregulated. Other metabolites that may be connected to uric acid excretion, purine metabolism, and inflammatory responses are also altered [9]. Among these metabolites, intestinal epithelial cells can use the energy from acetate, succinate, and glucose to support uric acid excretion and so reduce hyperuricemia [73].

Since a large number of transporters are secreted by naturally occurring bacteria in the human gastrointestinal tract, numerous transporters that act as mediators in uric acid absorption or secretion in the intestinal tract have been found [74]. These transporters arbitrate crucial processes in gut microbiota metabolism. The primary urate secretion transporter that mediates intestinal urate excretion and controls human serum uric acid levels is the ATP-binding cassette subfamily G2 (ABCG2), which is found in several regions of the small and large intestines [75]. Another important transporter that helps regulate uric acid is glucose transporter 9 (GLUT9, sometimes referred to as SLC2A9). Hyperuricemia and other metabolic syndrome symptoms may arise from intestinal SLC2A9 mutations or knockdowns. It is also known that other transporters, including SLC16A9, SLC17A4, SLC17A1, SLC17A3, SLC22A11, SLC22A12, and SLC16A9, are involved in the control of uric acid [76, 77].

### 3.7. Gut Microbiota and Hyperglycaemia

Hyperglycaemia is defined as having blood glucose levels higher than 125 mg/dL when fasting and 180 mg/dL two hours after a meal. One feature of metabolic syndromes is hyperglycaemia, which is strongly associated with distortions in the makeup of the gut microbiota. A microbiota investigation of four male Zucker diabetic fatty rats was recently carried out [78]; according to their findings, alterations in faecal microorganisms are associated with the advancement of age and illness. The predominant phyla, comprising *Actinomicrobiota*, *Firmicutes*, *Bacteroidetes*, and *Proteobacteria*, were the predominant microbiota found in rat faeces at all-time points ranging from eight to fifteen weeks. However, among rats aged 8 to 10 weeks, *Lactobacillus* and *Turicibacter* were the most common genera. In rats that were 15 weeks old, the most prevalent species were *Bifidobacterium*, *Lactobacillus*, *Ruminococcus,* and *Allobaculum*. Animals with type 2 diabetes mellitus have a mild dysbiosis of the gut microbiome in terms of ecological ecology. In particular, butyrate can be used as a source of energy for colonocytes and can increase satiety. It can also effectively reduce inflammation, reduce carcinogenesis, reduce oxidative stress, and improve gut barrier function. In patients with type 2 diabetes mellitus, however, the numbers of some metabolically beneficial microbiota are reduced, such as butyrate-producing bacteria, while the number of pathogenic bacteria that are known to cause various other conditions is increased [79]. Several studies have found a significant association between hyperglycaemia and dysbiosis of the gut microbiota, but the results were inconsistent, emphasizing the need for more research [80].

### 3.8. Gut Microbiota and Therapeutic Opportunities

The possibility of using microbiota manipulation to treat diseases has gained traction as our understanding of bacteria has grown. Since the human gut is involved in many different physiologic processes, it is anticipated that altering it will either prevent or treat the corresponding disorders. Consequently, a number of clinical trials are being conducted to look at this potential.

### 3.9. Modulations of the Microbiota: Probiotics, Prebiotics, and Symbiotics

A prebiotic is a substrate that is selectively utilized by host microorganisms to confer a health benefit. Fermentable fibres, including inulin, fructooligosaccharides, and galactooligosaccharides, are among the most frequently utilized prebiotics. All of these prebiotics have been tested to see whether they can help with metabolic diseases [81]. Insulin resistance is improved in overweight or obese people by inulin or inulin-propionate ester supplementation [82]. Additionally, a meta-analysis revealed that inulin-type fructans enhanced the assessment of insulin resistance in prediabetic and type 2 diabetic patients using the homeostasis model, fasting insulin, glycosylated haemoglobin, and fasting blood glucose [19]. Prebiotics may help with metabolic diseases, according to research; nevertheless, not many prebiotic health claims have been approved.

Probiotics are live microorganisms that, when administered in adequate amounts, confer a health benefit on the host. Probiotics that are frequently used include *Bifidobacterium* (*adolescentis*, *animalis*, *bifidum*, *reuteri*, *breve*, and *longum*) and *Lactobacillus* (*acidophilus*, *casei*, *fermentum*, *gasseri*, *johnsonii*, *paracasei*, *plantarum*, *rhamnosus*, and *salivarius*). Using mainly species from the genera *Lactobacillus* and *Bifidobacterium*, several randomized placebo-controlled trials have been conducted in type 2 diabetic subjects, and meta-analyses indicate that probiotic supplementation can improve glucose homeostasis in patients with type 2 diabetes [83]. The administration of live bacteria to the distal colon presents certain difficulties; nonetheless, better delivery and engraftment might increase the beneficial outcomes. The bacteria must first endure harsh circumstances, such as exposure to oxygen, low stomach pH, bile acids, and enzymes, while being stored and moving through the digestive system. The live bacteria can be shielded from these hostile environments and have their viability increased by using microencapsulation [84].

The term “symbiotic” refers to a combination of live microorganisms and substrates that the host microorganisms specifically use to promote the host's health. The ingestion of symbiotics may be necessary for the bacterial engraftment in the intestine that is necessary for the probiotics to have their therapeutic effects. Recently, individuals with type 2 diabetes have been investigated for this kind of symbiotic treatment [85].

### 3.10. Faecal Microbiota Transplantation (FMT)

This refers to a procedure whereby a recipient's digestive tract is filled with a donor's faecal matter solution in an attempt to treat their illness. This application of FMT treatment dates back to the 4th century in China [86]. This method will alter the recipient's microbial composition immediately. With reported cure rates close to 90%, recurrent *Clostridium difficile* infection has been the most notable outcome of the use of faecal transplantation for disease therapy. The many FMT techniques include colonoscopy, gastroscopy, and the use of a naso-intestinal tube, each with varying degrees of effectiveness. It has been established by multiple meta-analyses that FMT, being minimally invasive, is better than conventional antibiotic treatment [87].

### 3.11. Engineering Gut Bacteria

Modern DNA technology has made it possible to engineer bacteria for the treatment of disease. Based on conventional genetic engineering techniques, engineered probiotics have been used to treat colitis, diabetes, obesity, and a variety of pathogenic infections [88]. *Lactobacillus jannaschii*, a conventional female vaginal flora, has been modified to secrete HIV-resistant cyanovirin-N protein; this engineered bacterium has been shown to reduce HIV infection in rhesus monkeys by 63%. Other types of engineered bacterial therapies for diseases include synthetic immune regulatory proteins, chemotactic response systems, and protein delivery systems [89].

### 3.12. Psychobiotics

The class of drugs known as “psychobiotics” includes probiotic, postbiotic, prebiotic, and symbiotic medicines that affect the gut-brain axis and improve mental health. Stress, anxiety, and sadness can all be psychotropically affected by psychobiotics via immunoregulatory pathways, the neuroendocrine system, and vagus nerves, with which the brain and gut microorganisms can communicate [90]. Psychobiotics function by altering the pathways involved in cognition and emotion, by focusing on the hypothalamic-pituitary-adrenal axis for inflammatory molecules that are directly linked to depression, or by focusing on proteins and neurotransmitters that are involved in brain function. Human microorganisms that boost interleukin-10, such as *Lactobacillus* GG and *Bifidobacterium infantis*, contribute to the preservation of the blood-brain barrier by either directly or indirectly lowering proinflammatory cytokines. In a similar vein, spore-forming human gut microbes have been shown to enhance serotonin biosynthesis in enterochromaffin cells. Consequently, psychobiotics have become a viable treatment option for a number of neurodegenerative disorders and can be a helpful and promising approach to overall health. Despite the encouraging results, there are currently insufficient human studies on the subject, and more research is needed to establish psychobiotics as an alternative therapy for neurodevelopmental and neurodegenerative disorders [91].

### 3.13. Bacteriophages

These viruses are unique to a certain type of bacteria. Bacteriophages are employed to target antibiotic-resistant infections because they promote bacterial membrane disintegration by inserting their genome within the targeted organism. Antibiotic-resistant microbial infections are the source of many diseases that are treated using phage and phage products [92]. Phages are host-specific; they only affect their target hosts and have no negative effects on the host environment. Additionally, there are multiple ways to inject the phage, which makes treatment easier. Furthermore, phage has the ability to mutate in order to stop the host from developing resistance. Phage treatment has many disadvantages, notwithstanding the benefits. With CRISPR-Cas adaptive immunity, spontaneous mutations, or restriction modification, bacteria can become resistant to phage. The accurate identification of the bacterial pathogen should come before phage therapy. Phage treatment has demonstrated no efficacy in certain circumstances. More so, because of the influence of gastric secretions, phage therapy requires a neutralized environment, which cannot be produced within the digestive tract [93].

### 3.14. Limitations of Gut Microbiota Therapeutics

Although microbiome therapies have a good record of accomplishment, they typically face some difficulties. Finding the most suitable bacteria to treat complex diseases is the main obstacle in the field of microbiome therapies [94]. The biogeography of the illness determines which probiotic is best suited for treatment. It is necessary to characterize microorganisms according to their functional advantages before selecting them for treatment. For a considerable amount of time and in a variety of situations, the effectiveness of microbiome therapies has been problematic. Furthermore, human trials for microbiome therapeutics are still a work in progress, with the majority of the research conducted on rodent models. Chemostats have to be constructed in order to fully understand the environmental circumstances that microorganisms confront as well as the interactions between microbes that influence their functions [95].

To ensure that clinical trials of microbiome therapies are successful, a number of safety and regulatory concerns must be investigated. A regulatory framework must address the biosafety of therapies to lessen side effects and the discharge of modified germs into the environment. It is necessary to evaluate the safety of modified probiotics in order to ensure long-term therapeutic efficacy. One of the main concerns is the potential horizontal transfer of recombinant DNA from the designed microbiome to the natural microbiome [96]. In a similar vein, recombinant probiotics released into the environment may be harmful [97]. Because they cannot colonize external habitats, auxotrophic bacteria that become viable in the absence of a specific substrate must be employed as treatments. Because modified phage may result in their loss of function, coordinated research and regulatory mechanisms are therefore necessary for both therapeutic maintenance and a safe therapeutic strategy [98].

## 4. Conclusion and Future Perspectives

Based on available clinical and experimental data, gut microbes may have a pathogenic role in the emergence of metabolic disorders. Keeping up with the most recent scientific findings and technological advancements in this ever-evolving subject is essential to realizing the full potential of comprehending and modifying the gut microbiota for the improvement of human health. Problems like individual variability and microbiological complexity continue to exist and call for ongoing research and technological breakthroughs. Future interventions could involve integrating microbiome data with genomics, transcriptomics, and metabolomics for a more thorough understanding of host-microbiota interactions, as well as using artificial intelligence and machine learning to more effectively analyse large-scale microbiome data sets and identify recurring patterns. Subsequent research endeavours could reveal distinct microbial signatures linked to different elements of metabolic syndromes. This would establish a basis for precision medicine in this field, ultimately augmenting healthcare quality and cultivating a more tailored comprehension of human biology.

## Data Availability

All data used are presented in the review article.

## Abbreviations

SCFAs: Short-chain fatty acids
BMI: Body mass index
TMA: Trimethylamine
FMO3: Flavin-containing monooxygenase 3
TMAO: Trimethylamine N-oxide
FFAR: Fatty acid receptor
ABCG2: ATP-binding cassette subfamily G2
FMT: Faecal microbiota transplantation.
